# Abnormal Cytokine Profile in Patients with Obstructive Sleep Apnea-Hypopnea Syndrome and Erectile Dysfunction

**DOI:** 10.1155/2014/568951

**Published:** 2014-05-19

**Authors:** Izolde Bouloukaki, Vaios Papadimitriou, Frank Sofras, Charalampos Mermigkis, Violeta Moniaki, Nikolaos M. Siafakas, Sophia E. Schiza

**Affiliations:** ^1^Sleep Disorders Center, Department of Thoracic Medicine, University of Crete, 711 10 Heraklion, Crete, Greece; ^2^Department of Urology, University of Crete, 711 10 Heraklion, Crete, Greece

## Abstract

Patients with obstructive sleep apnea-hypopnea syndrome (OSAHS) show a high prevalence of erectile dysfunction (ED). Although the underlying pathogenesis is still unknown, endothelial dysfunction, induced by inflammatory cytokines, chemokines, and adhesion molecules, has been proposed as a possible mechanism. The aim of this study was to assess whether OSAHS is associated with activation of the inflammatory cytokine system in patients with ED compared to the matched OSAHS patients with normal sexual function. Thirty-one patients with severe OSAHS and ED were included. Fifteen patients with severe OSAHS and without ED served as controls. Serum concentrations of high-sensitivity C-reactive protein (hsCRP), tumor necrosis factor-**α** (TNF-a), interleukin-6 (IL-6), interleukin-8 (IL-8), and adiponectin were measured after the diagnostic polysomnography. We found that hsCRP levels were significantly elevated in OSAHS patients with ED compared to controls. Similarly, TNF-a levels, IL-6, and IL-8 were elevated in OSAHS patients with ED compared to controls. Serum adiponectin levels were lower in OSAHS-ED patients, but the difference did not reach statistical significance. The presence of ED in patients with severe OSAHS is associated with elevated levels of inflammatory markers, underlining a possible involvement of endothelial dysfunction in the pathogenesis of ED.

## 1. Introduction

Obstructive sleep apnea-hypopnea syndrome (OSAHS) is a common disease characterized by repetitive episodes of partial or complete upper-airway obstruction during sleep. Although estimates of disease prevalence are in the range of 3–7% for adult men and 2–5% for adult women in the general population [1], with prevalence rates reaching up to 33% in certain populations [2], OSAHS tends to be underdiagnosed in clinical practice [3]. OSAHS is associated with various neurobehavioral and cardiovascular sequelae, among which sexual dysfunction remains the least studied. However, erectile dysfunction (ED) is a highly prevalent condition in patients with OSAHS [4, 5], and its frequency and severity appear to correlate with the severity of OSAHS [6]. In addition, up to 91% of patients with erectile dysfunction may be diagnosed with OSAHS [7].

ED has been defined as the persistent inability to attain and maintain an erection sufficient to permit satisfactory sexual performance [8]. The prevalence of ED is also high, varying in different countries between 3% and 71% according to age [9]. In Greece, Doumas and colleagues reported an ED prevalence of 14.1% in normotensive patients compared to 35.2% in patients with essential hypertension [10]. There is a growing body of evidence suggesting that ED is predominantly a disease of vascular origin, with endothelial cell dysfunction as the unifying link [11]. Circulating markers of endothelial cell damage that have previously been reported include cytokines and chemokines, soluble adhesion molecules, and acute-phase reactants. Several inflammatory cytokines, such as interleukin-1 (IL-1), interleukin-6 (IL-6), tumor necrosis factor-a (TNF-a), and high-sensitivity C-reactive protein (hsCRP), have been investigated and could be proposed as laboratory markers for potential use in ED [12]. Moreover, in vitro studies demonstrate that the above inflammatory factors suppress the production of adiponectin, which is released from adipose cells and exerts a variety of anti-inflammatory effects [13].

The underlying mechanisms by which OSAHS and ED interact are still unknown. Several mechanisms have been proposed, including nerve involvement caused by hypoxemia, low levels of testosterone, and vascular endothelial dysfunction through diverse pathways such as hypoxemia, oxidative stress, and sympathetic activation [14]. There is convincing evidence for endothelial dysfunction in OSAHS; however, there are contradictory findings if administration of continuous positive airway pressure (CPAP) reverse endothelial dysfunction associated with OSAHS [15, 16]. Endothelial dysfunction, as already mentioned, is also a key finding in the patient with ED [11], suggesting a mechanistic paradigm whereby OSAHS may lead to ED. However, limited information exists regarding the pathophysiologic basis of ED in OSAHS, in particular, regarding how intermittent nocturnal hypoxemia is likely to contribute to impaired penile tumescence by promoting endothelial dysfunction, which might be mediated by elevated levels of inflammatory markers as well as decreased levels of anti-inflammatory markers. Therefore, the aim of this study was to assess whether OSAHS is associated with activation of the inflammatory cytokine system in patients with ED compared to patients with OSAHS and normal sexual function, evaluating a wide range of inflammatory (hsCRP, IL-6, IL-8, and TNF-a) and anti-inflammatory (adiponectin) markers.

## 2. Materials and Methods

### 2.1. Study Population

From June 2011 to June 2012, patients were recruited from a prospective study cohort comprising consecutive patients, between 18 and 65 years old, who visited the Sleep Disorders Center, Department of Thoracic Medicine, Medical School University of Crete, for evaluation of suspected sleep-disordered breathing. The inclusion criteria were the following: newly diagnosed OSAHS by polysomnography (PSG) according to standard criteria, severe OSAHS, and an above-elementary school education. The exclusion criteria were the following: refusal to participate, previous CPAP treatment, hypertension, coronary artery disease, congestive heart failure, history of life-threatening arrhythmias, cardiomyopathy, history of stroke, chronic renal disease, endocrine dysfunction, such as diabetes mellitus, Cushing's syndrome, and abnormal pituitary function, chronic obstructive pulmonary disease, morbid obesity (BMI > 40 kg/m^2^), dyslipidemias, undertreatment with cardiovascular medication, or pharmacologically treated depression, with severe cognitive impairment, with a family or personal history of mental illness, with drug or alcohol abuse, with concurrent oncologic diseases, metabolic or neurological disorders known to induce peripheral neuropathy or ED, deep vein thrombosis, peripheral vascular disease, connective tissue disorders, and history of narcolepsy. Moreover, if patients were diagnosed with ED before the enrolment of this study or had already undergone therapy with medications that affect erectile function, such as b-blockers and H_2_ blockers, they were also eliminated from the study. All subjects provided written informed consent, and ethical approval was provided by the University Hospital Ethics Committee.

### 2.2. Data Collection: Study Design

The study subjects underwent a detailed evaluation that included age (years), body mass index (BMI) (kg/m^2^), clinical history focused on sleep-related symptoms, associated conditions and comorbidities, medication use, smoking history, and alcohol intake. Furthermore, a urology specialist assessed sexual history and analyzed the use of medications that may interfere with erection function. Each patient was asked to complete the Greek version of the validated International Index of Erectile Function (IIEF) Questionnaire as a comprehensive tool for assessing erectile function [17]. In addition, the Epworth Sleepiness Scale (ESS) was used to assess the degree of diurnal somnolence [18].

After signing the informed consent form, the PSG was scheduled. The patients arrived at the Sleep Disorders Center at least 2 hours before their normal bedtime and went to sleep at their habitual bedtimes, and blood samples were collected the following morning for biochemical and haematological analyses. A sleep physician coordinated any necessary clinical followup and made the results of the exams available for each patient. During these appointments, they received one-on-one counselling by a sleep physician regarding the results of their PSG studies, basic information on OSAHS, its known effects on comorbid conditions, proper sleep hygiene, adjunctive/conservative methods to improve sleep, and the importance of treatment adherence.

### 2.3. Questionnaires

#### 2.3.1. IIEF

The IIEF is divided into five domains (erectile function, intercourse satisfaction, orgasm, sex drive, and overall satisfaction). The erectile function domain consists of six questions (questions 1 to 5 and question 15) and has a maximum score of 30 and a minimum score of 6. Each question is scored on a 5-point Likert scale, with 5 representing the best score. A score of less than 26 is indicative of ED [17].

#### 2.3.2. ESS

The ESS is currently the most used subjective test of daytime sleepiness in clinical practice. It is a simple, self-administered, and eight-item questionnaire that measures the risk of falling asleep in eight situations that are commonly met. Total score >10 is considered excessive daytime sleepiness. The higher the score (from 10 to 24), the greater the reported subjective daytime sleepiness [18].

### 2.4. Polysomnography

All patients underwent a single-night full diagnostic PSG (Alice 4, 5, Diagnostics System, Respironics, USA) according to standard techniques, with monitoring of the electroencephalogram (EEG) using frontal, central, and occipital leads, electrooculogram (EOG), electromyogram (EMG), flow (by oronasal thermistor and nasal air pressure transducer), thoracic and abdominal respiratory effort (induction plethysmography), oximetry, and body position. Snoring was recorded by a microphone placed on the anterior neck. Polysomnographic recordings were manually interpreted over 30-second periods, in accordance with the 2007 guidelines of the American Academy of Sleep Medicine (AASM) [19]. The scorer was always the same person, blinded to the clinical condition of the patients and the previous results of the questionnaires. The determination of sleep stages and arousals was performed according to the AASM 2007 criteria using EEG montages, including frontal, central, and occipital leads [19].

Apnea was defined as a cessation of airflow (≥90%) for at least 10 seconds, and hypopnea was defined as a ≥30% reduction of airflow (from the nasal pressure transducer signal) lasting at least 10 seconds with ≥4% desaturation. The Apnea-Hypopnea Index (AHI) was calculated as the number of apnea and hypopnea events per hour of sleep.

### 2.5. Blood Collection and Analysis

Shortly after the conclusion of the overnight sleep recordings, venous blood was collected from all subjects between 8:00 a.m. and 9:00 a.m., following an overnight fast, for measurement of hsCRP, TNF-a, IL-6, IL-8, and adiponectin. All venous samples were centrifuged within 30 minutes at 3000 rpm for 15 min, and serum was separated into multiple aliquots and stored at −80°C until analysis. hsCRP levels, expressed in mg/dL, were measured by particle-enhanced immunonephelometry using BN Systems (Dade Behring Inc.; Newark, USA). Quantitative measurements of TNF-a, IL-6, and IL-8 in serum were made using an automated chemiluminescence analyzer (Immulite 1000, DPC) with reagents from the same manufacturer. For these three markers, the results were expressed as pg/mL. Serum levels of adiponectin were determined using a quantitative sandwich enzyme immunoassay technique (Quantikine Human Adiponectin Immunoassay, R&D Systems), which measures total human adiponectin (low, middle, and high molecular weight forms). The results were expressed as ng/mL.

### 2.6. Statistical Analysis

Values are expressed as mean (SD) or median value with interquartile range (IQR), depending on the data distribution. Comparisons between groups were performed using the nonparametric Mann-Whitney *U* test. The degree of OSAHS, assessed by the AHI, in the whole group and separately in patients with OSAHS-ED and controls was correlated with the levels of CRP and other cytokines using Pearson's correlation analysis. *P* < 0.05 was considered the threshold for statistical significance. All statistics were calculated using the Statistical Package for Social Sciences software, version 17.0.0 (SPSS, Chicago, Illinois, USA).

## 3. Results

During the study period, 1034 patients underwent diagnostic PSG for a suspected sleep disorder. Of them, 404 patients volunteered to participate in this study and completed the IIEF Questionnaire. Most of the participants were middle aged (42.6 ± 9.3 years old) and moderately obese (BMI: 33.3 ± 5.05 Kg/m^2^). The overall ED prevalence, based on IIEF score, was 40.9% (165 patients) of the study population. The patients with severe OSAHS had higher incidence of ED compared with those moderate or mild OSAHS patients (53.5% versus 27.1%; *P* = 0.005). The mean IIEF score was significantly lower in patients with severe OSAHS (23.84 ± 6.38 versus 26.1 ± 4.9, *P* = 0.001). Of the 404 patients, most of them were not eligible for further evaluation because of the presence of at least one of the exclusion criteria. Finally, 31 patients with severe OSAHS who suffered from ED (IIEF < 26) and met the inclusion criteria agreed to participate in the study and were available for analysis. Fifteen patients with normal erectile function, who had similar age, BMI, and AHI distributions to the study group, served as controls.  Table 1 shows the baseline demographic and PSG characteristics of the ED patients and controls. Apart from their IIEF scores, there were no statistically significant differences between the two groups.

**Figure 1:**
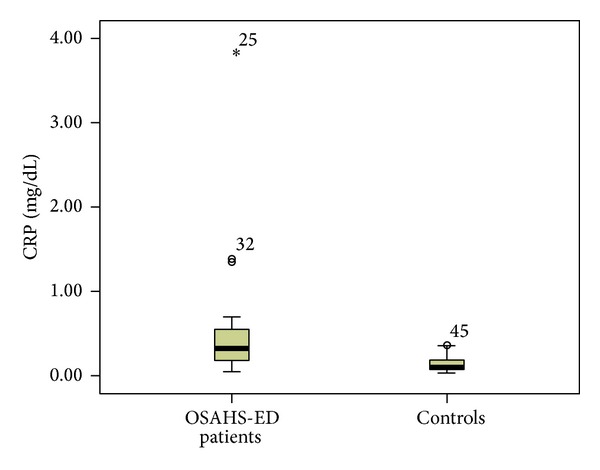
C-reactive protein (CRP) levels (mg/dL) are significantly higher in OSAHS patients with ED (OSAHS-ED) compared to controls (*P* < 0.001). CRP concentrations are shown as median (interquartile range) in the boxes.

**Figure 2:**
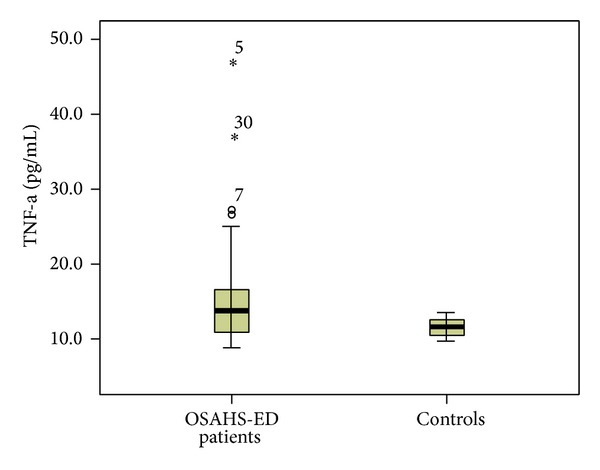
Tumor necrosis factor-*α* (TNF-a) levels (pg/mL) are significantly higher in OSAHS patients with ED (OSAHS-ED) compared to controls (*P* = 0.01). TNF-a concentrations are shown as median (interquartile range) in the boxes.

**Figure 3:**
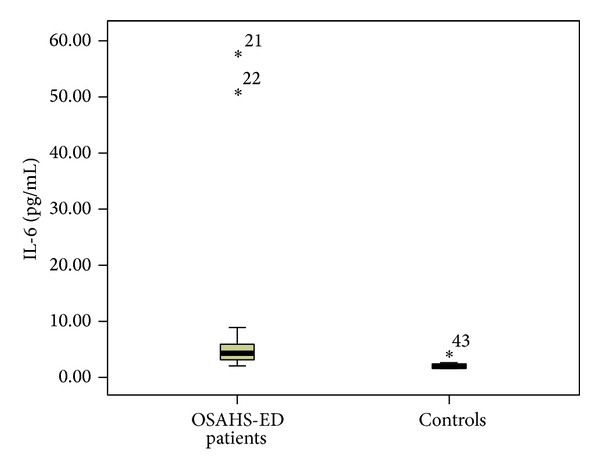
Interleukin-6 (IL-6) levels (pg/mL) are significantly higher in OSAHS patients with ED (OSAHS-ED) compared to controls (*P* < 0.001). IL-6 concentrations are shown as median (interquartile range) in the boxes.

**Figure 4:**
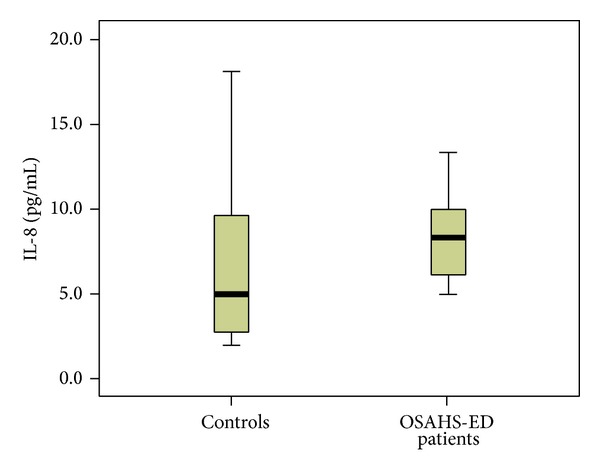
Interleukin-8 (IL-8) levels (pg/mL) are significantly higher in OSAHS patients with ED (OSAHS-ED) compared to controls (*P* = 0.034). IL-8 concentrations are shown as median (interquartile range) in the boxes.

**Figure 5:**
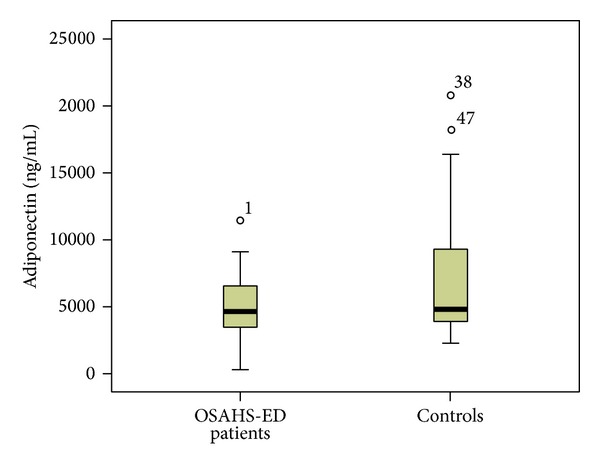
Adiponectin levels (ng/mL) were lower in OSAHS patients with ED (OSAHS-ED) compared to controls, but the difference did not reach statistical significance (*P* = 0.5). Adiponectin concentrations are shown as median (interquartile range) in the boxes.

Patients with ED had significantly higher median (interquartile range (IQR)) plasma levels of hsCRP [0.32 (0.38) versus 0.1 (0.17) mg/dL, *P* < 0.001] than controls (Figure 1). TNF-a was significantly elevated in ED patients compared to controls [13.8 (5.8) versus 11.55 (2.2) pg/mL, *P* = 0.01] (Figure 2). Furthermore, significantly higher levels of IL-6 [4.38 (2.74) versus 2 (0.39) pg/mL, *P* < 0.001] (Figure 3) and IL-8 [8.29 (5.2) versus 4.98 (7.0) pg/mL, *P* = 0.034] were observed in ED patients (Figure 4). Adiponectin levels were lower in OSAHS-ED patients, but the difference did not reach statistical significance [4680.6 (3154) versus 4864.6 (8418) ng/mL, *P* = 0.5] (Figure 5).

The degree of OSAHS in the whole group assessed by the AHI showed a statistical significant correlation only with CRP values (*P* = 0.013 and *r* = 0.52) and not with the other measured cytokines. It is notable that the significant association between OSAHS severity and CRP was observed only in OSAHS patients with ED (*P* < 0.001, *r* = 0.896 and *P* = 0.013, *r* = 0.52, OSAHS patients with and without ED, resp.).

## 4. Discussion

Sexual dysfunction represents a significant health problem and may have a strong negative impact on the quality of life. Therefore, the identification of potentially modifiable risk factors, such as OSAHS, may be important for disease prevention and treatment. Although ED is a frequent occurrence in male patients with OSAHS, the precise mechanisms mediating this morbidity are currently unknown.

The present study is the first to investigate low-grade inflammation and an altered endothelial state in patients with ED and severe OSAHS by evaluating a wide spectrum of circulating markers and mediators. With a view to explaining the association between ED and OSAHS, patient selection was strict enough to exclude the presence of other comorbidities that are also known as risk factors for ED. Our results showed that the combination of ED and OSAHS was associated with higher levels of inflammatory markers compared to OSAHS alone.

Previous studies have shown that ED could be a disease of low-grade inflammation [20, 21]. Vlachopoulos et al. [21] demonstrated an increase in the levels of inflammatory markers such as hsCRP, IL-6, IL-1*β*, and TNF-a in patients with ED, suggesting that low-grade systemic inflammation is present in these subjects, similar to that seen in insulin resistance, obesity, type 2 diabetes mellitus, hypertension, hyperlipidaemia, and metabolic syndrome X. Moreover, other investigators reported that ED was associated with increased levels of TNF-a and CRP, which increased progressively with the severity of penile vascular disease, supporting the role of these markers in the pathophysiology of ED [22, 23]. Furthermore, recently, Matos et al. showed that there was an association between TNF-a levels and ED complaints in men independent of OSAHS [24]. It is worth noting that, in a recent study, reduced levels of adiponectin, an anti-inflammatory cytokine that attenuates endothelial cell adhesion molecules, and the levels of inflammatory cytokines, such as TNF-a, IL-8, and IL-6, were observed in patients with ED [25].

There are several explanations regarding why this link between ED and OSAHS exists, implicating hormonal, neural, and endothelial mechanisms. An important link connecting ED and OSAHS is endothelial dysfunction. Patients with OSAHS tend to have lower levels of nitric oxide (NO), which is responsible for vasodilatation and erection [26]. NO suppresses the production of TNF-a and IL-1. Similarly, oxidative stress, one of the promoters of endothelial dysfunction, is enhanced in OSAHS, promoting ED. The combination of repetitive hypoxemia and sleep deprivation in OSAHS patients may be associated with NO deficiency, resulting in increased levels of well-known inflammatory cytokines, such as IL-6, IL-8, adhesion molecules, and hsCRP [27, 28]. Increased expression of these inflammatory cytokines may contribute to endothelial dysfunction, which may cause ED [14]. Indeed, a number of studies have demonstrated a causal relationship between OSAHS and endothelial dysfunction, which was improved or not by CPAP treatment [16, 29, 30]. A chronic hypoxic condition also contributes to low levels of adiponectin; however, there are conflicting opinions regarding adiponectin levels in patients with OSAHS. Although a few studies reported that adiponectin was more strongly correlated with AHI in patients who had OSAHS, compared with various other factors such as age and obesity [31–34], other studies have reported that adiponectin levels were largely unaffected by the syndrome [35–37].

The elevated markers of endothelial damage and decreased anti-inflammatory adiponectin found in our study underline the involvement of endothelial dysfunction in the pathogenesis of ED, which also comprises a pathophysiological link between this entity and OSAHS. Considering OSAHS as a chronic disease state, it must play a role in the development of ED, leading to subsequent overall dissatisfaction with sexual function. However, as the two groups of patients were matched for severity of OSAHS, one could argue why one group would experience the elevated cytokine levels and ED while the other group was spared. Possibly, the disparity in responses among the two groups is based on the heterogeneity of the magnitude of end-organ morbidity in sleep apnea among patients and shows that not everyone will be affected to the same extent. Furthermore, the observation that treatment for sleep apnea restores erectile function argues for a direct role of OSAHS in the pathogenesis of ED [38, 39]. Therefore, OSAHS could be considered as a sensitive predictor for ED. These novel findings clearly warrant further research aimed at defining the roles of inflammatory markers and associated factors, such as testosterone levels and hormonal disorders, in subjects who are at high risk of ED in the early detection of low-grade systemic inflammation and in the prevention, prediction, and prognosis of ED. Subsequently, larger scale studies should be conducted and performed including subjects with comorbidities and various age groups. Screening and multidisciplinary approach must be adopted in patients at risk of ED, and there is a need not only for clinical, but also for public health interventions.

The present study had some limitations that deserve comment. Firstly, the analysis was conducted on a small population and not based on a power calculation, depriving the ability to conduct more robust statistical methods. This was due to the difficulty of including only middle-aged patients with newly diagnosed OSAHS who had no comorbidities and were not under treatment with cardiovascular medication. In addition, a high refusal rate limited the patients' participation and may have been related to embarrassment at reporting such symptoms. Secondly, ED was assessed based exclusively on the IIEF Questionnaire, a self-reported, subjective estimation of erectile function in men, and not on objective measures, such as nocturnal penile tumescence, testosterone measurements. However, this questionnaire has been validated by multiple studies for evaluating erectile function in various patient populations and has been shown to be clinically appropriate for the evaluation of erectile function; therefore, it can serve as an indirect indicator of ED. Thirdly, we included only patients with severe OSAHS, so further studies are necessary in order to investigate possible relations between ED and cytokine profile in OSA patients with moderate or even mild disease. Finally, patients with other additional risk factors for ED were excluded, which does not represent well the situation found in clinical practice. However, as per Huang et al., subclinical endothelial dysfunction may underlie organic ED in young patients without well-known related risk factors [40].

## 5. Conclusions

In conclusion, our results showed that the presence of ED in patients with severe OSAHS is associated with higher levels of inflammatory markers and lower levels of an anti-inflammatory marker, adiponectin, compared to patients with OSAHS of the same severity but without ED. The increased markers of endothelial damage underline a possible involvement of endothelial dysfunction in the pathogenesis of ED.

## Figures and Tables

**Table 1:** Baseline clinical and PSG characteristics of controls and OSAHS patients with ED (OSAHS-ED).

	Controls (*n* = 15)	OSAHS-ED patients (*n* = 31)
Age (years)	47.9 ± 7.9	48.5 ± 8.55
IIEF	28.1 ± 1.2	14.35 ± 6.6*
BMI (kg/m^2^)	32.6 ± 3.21	32.4 ± 3.74
AHI (events/h of sleep)	47.82 ± 23.56	48.09 ± 26.39
ODI	30 ± 18	46 ± 22
TST (min)	313 ± 95	274 ± 75
SE (%)	71 ± 16	81 ± 55
AI	42 ± 8	44 ± 17
NREM (min)	291 ± 91	255 ± 66
SWS (min)	25 ± 16	23 ± 10
REM (min)	24 ± 16	23 ± 13
Mean SaO_2_ (%)	92.3 ± 3.87	92.5 ± 3.6
Minimum SaO_2_ (%)	80.14 ± 8.91	79.32 ± 9.68

Values are mean ± (SD).

PSG: polysomnography; OSAHS: obstructive sleep apnea-hypopnea syndrome; ED: erectile dysfunction; IIEF: International Index of Erectile Function Questionnaire; BMI: body mass index; AHI: Apnea-Hypopnea Index; ODI: oxygen desaturation index; TST: total sleep time; SE: sleep efficiency; AI: Arousal Index; SaO_2_: oxygen saturation; SWS: slow wave sleep.

*P < 0.001.
